# Extracellular excystation and development of *Cryptosporidium*: tracing the fate of oocysts within *Pseudomonas* aquatic biofilm systems

**DOI:** 10.1186/s12866-014-0281-8

**Published:** 2014-11-18

**Authors:** Wan Koh, Andrew Thompson, Hanna Edwards, Paul Monis, Peta L Clode

**Affiliations:** School of Veterinary and Life Sciences, Murdoch University, South Street, Murdoch, WA 6150 Australia; South Australian Water Corporation, 250 Victoria Square, Adelaide, SA 5000 Australia; Centre for Microscopy, Characterisation and Analysis, The University of Western Australia, 35 Stirling Hwy, Crawley, WA 6009 Australia; School of Occupational and Public Health, Ryerson University, 350 Victoria Street, Toronto, Ontario M5B2K3 Canada

**Keywords:** *Cryptosporidium*, Biofilms, Extracellular multiplication, Excystation, Confocal microscope, Scanning electron microscope, Flow cytometry

## Abstract

**Background:**

Aquatic biofilms often serve as environmental reservoirs for microorganisms and provide them with a nutrient-rich growth environment under harsh conditions. With regard to *Cryptosporidium*, biofilms can serve as environmental reservoirs for oocysts, but may also support the growth of additional *Cryptosporidium* stages.

**Results:**

Here we used confocal laser scanning microscopy, scanning electron microscopy (SEM), and flow cytometry to identify and describe various *Cryptosporidium* developmental stages present within aquatic biofilm systems, and to directly compare these to stages produced in cell culture. We also show that *Cryptosporidium* has the ability to form a parasitophorous vacuole independently, in a host-free biofilm environment, potentially allowing them to complete an extracellular life cycle. Correlative data from confocal and SEM imaging of the same cells confirmed that the observed developmental stages (including trophozoites, meronts, and merozoites) were *Cryptosporidium*. These microscopy observations were further supported by flow cytometric analyses, where excysted oocyst populations were detected in 1, 3 and 6 day-old *Cryptosporidium*-exposed biofilms, but not in biofilm-free controls.

**Conclusions:**

These observations not only highlight the risk that aquatic biofilms pose in regards to *Cryptosporidium* outbreaks from water distribution systems, but further indicate that even simple biofilms are able to stimulate oocyst excystation and support the extracellular multiplication and development of *Cryptosporidium* within aquatic environments.

**Electronic supplementary material:**

The online version of this article (doi:10.1186/s12866-014-0281-8) contains supplementary material, which is available to authorized users.

## Background

*Cryptosporidium* is one of the most common enteric protozoan parasites infecting both humans and animals [1] through water. Recently, the association of *Cryptosporidium* with biofilms in water bodies was shown to be responsible for frequent sporadic *Cryptosporidium* outbreaks [2,3]. Studies have also shown that *Cryptosporidium* oocysts incorporate readily into biofilms [4-6], and concerns have been expressed over the sudden sloughing of biofilms from water pipes with an accumulated aggregation or ‘bolus’ of oocysts that dramatically increase the infective dose needed to cause infection [7]. In addition, the association among biofilm communities is believed to influence the propagation of *Cryptosporidium* through both environmental and water treatment systems [8,9]. However, the behaviour of *Cryptosporidium* within a nutrient-rich biofilm is still not well defined [10,11].

As *Cryptosporidium* was believed to be an obligate intracellular parasite and, therefore, lacking the capability to proliferate within biofilms [12], initial studies focused only on the association of the oocyst stage within biofilms [5-7,9]. However, the notion that *Cryptosporidium* is an obligate intracellular parasite has been challenged following the observation of extracellular multiplication of *Cryptosporidium* in *in*-*vitro* and cell free cultures [13-15], and there is increasing evidence that *Cryptosporidium* is able to multiply extracellularly or in the absence of a host cell [14-18]. With this, quantitative polymerase chain reaction (qPCR) data have shown that *Cryptosporidium* can multiply within aquatic biofilms, with a 2–3 fold increase in *Cryptosporidium* numbers observed over a 6 day period [19].

To gain more detailed information about the dynamics and developmental biology of *Cryptosporidium* after exposure to biofilms, we have examined stages of *Cryptosporidium* produced in biofilms using confocal microscopy and scanning electron microscopy (SEM). Direct correlation between confocal and SEM data was performed (on the same cells) to both confirm that the observed structures were *Cryptosporidium* life stages, and to describe their structure in greater detail. Additionally, because excysted oocysts have a distinctly different morphology from that of intact oocysts and can be distinguished readily by flow cytometry [20-22], we have used flow cytometry to track morphological changes to oocysts after exposure to an artificial biofilm system.

## Results

### Confocal microscopy

**Figure 1:**
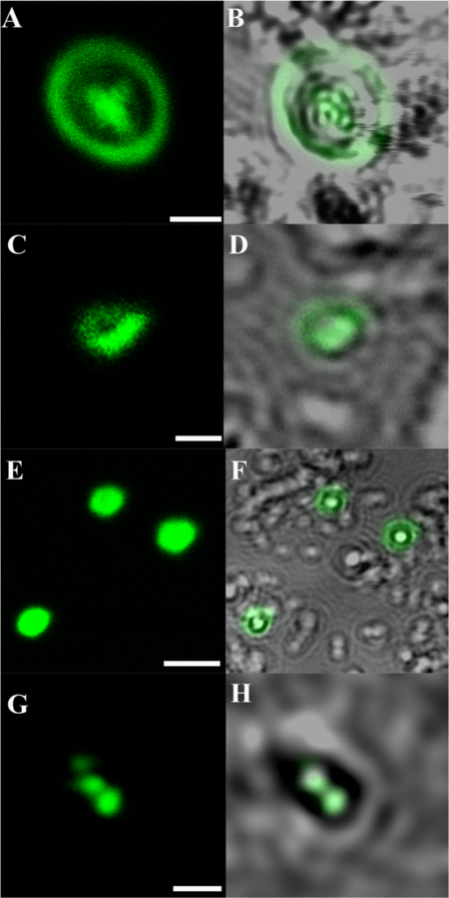
**Micrographs of** ***Cryptosporidium*** **within** ***Cryptosporidium-*** **exposed biofilms. (A & B)** Oocyst containing sporozoites; **(C & D)** Free sporozoites; **(E & F)** Individual trophozoites; **(G & H)** Aggregated trophozoites. **(A, C, E, G)** Confocal images; **(B, D, F, H)** Superimposed confocal and brightfield images. Scale bars = **A & G**: 3 μm; C: 1 μm; E: 4 μm.

**Figure 2:**
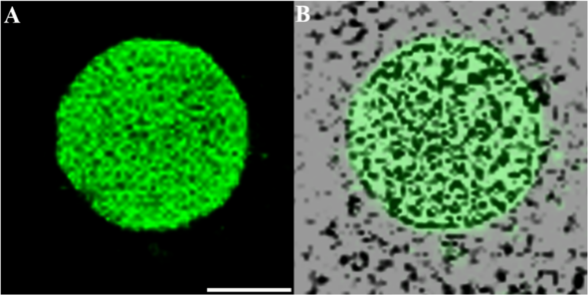
**Micrographs of a** ***Cryptosporidium*** **microgamont. (A)** Confocal image; **(B)** Superimposed confocal and brightfield images. Scale bar = 2 μm.

**Figure 3:**
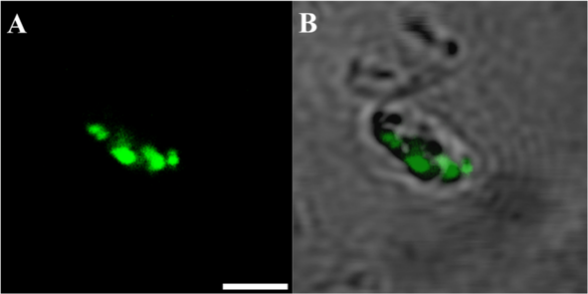
***Cryptosporidium*** **gamont**-**like cell identified within 6 day**-**old** ***Cryptosporidium***-**exposed biofilms. (A)** Confocal image; **(B)** Superimposed confocal and brightfield images. Scale bars = 2.5 μm.

From day 1, fluorescent oocysts containing internal sporozoites (Figure 1A and B) and free sporozoites (Figure 1C and D) with a rounded posterior and a pointed tapered anterior end were routinely observed. By day 3, well formed, individual trophozoites (3 × 3 μm: Figure 1E and F) were routinely observed, and occasionally, aggregations of trophozoites were also seen (Figure 1G and H). At day 6, microgamonts (Figure 2; 4.5 × 4.5 μm) presumably containing masses of microgametes were identified, confirming that the process of merogony had occurred within the aquatic biofilm environment. Interestingly, gamont-like cells were observed in the 6 day-old biofilms (Figure 3). This gamont-like stage in biofilms was also blunt-ended and rod-shaped (2 × 4 μm), which complements the description by Hijjawi et al. [14]. The internal structure of the cell was intensely labelled by Sporo-Glo™ antibody (Figure 3B).

### Scanning electron microscopy

**Figure 4:**
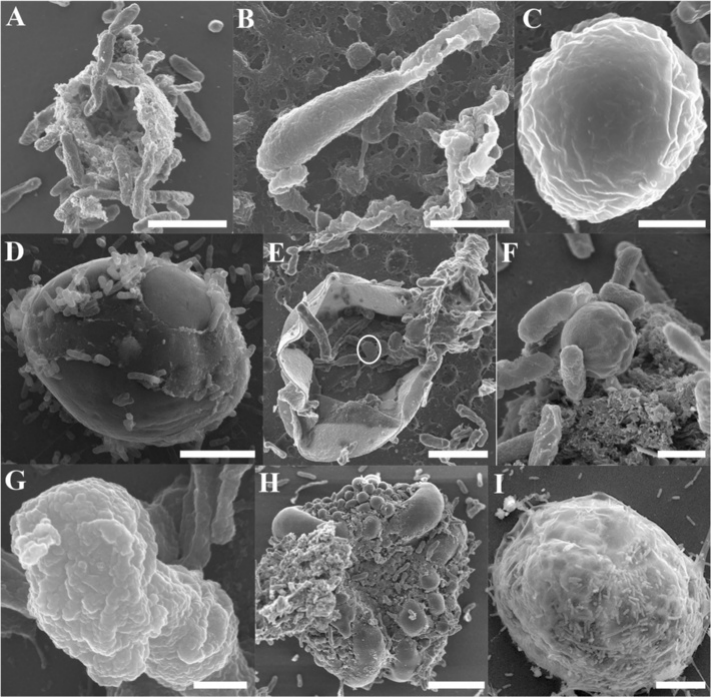
**Scanning electron micrographs of** ***Cryptosporidium*** **within** ***Cryptosporidium***-**exposed biofilms. (A)** Empty oocysts with a rough membrane appearance; **(B)** Free sporozoite; **(C)** Trophozoite; **(D)** Large gamont cells (meronts) identified within 6 day-old biofilms; **(E)** type II meront containing type II merozoites within (circled); **(F)** Free type I merozoites; **(G)** Free type II merozoites; **(H)** Microgamont; **(I)** Extra-large gamont. Scale bars = **A** & **B**: 2.5 μm; **C** & **F**: 1 μm; **D** & **E**: 3 μm; **G**: 500 nm; **H**: 5 μm; **I**: 8 μm.

In addition to confocal microscopy, high resolution imaging of *Cryptosporidium* extracellular stages was performed using SEM. Similar to our confocal microscopy observations, empty oocysts with a wide opening and rough membrane (Figure 4A) and free sporozoites (Figure 4B) were observed from day 1. By day 3, trophozoites (Figure 4C) and several large gamont-like cells (~10 μm; Figure 4D) similar in size to type I and II meronts observed previously by Koh et al. [19], were observed. These gamonts were further characterised as meronts when several unreleased type II merozoites were detected within a single type II meront (Figure 4E). Not surprisingly, free type I (circular shaped; 1.5 × 1.5 μm; Figure 4F) and II merozoites (spindle shaped; 1 × 2 μm; Figure 4G) were also frequently identified. The microgamonts within biofilms were also large (~15 μm; Figure 4H) and contained a large number of microgametes. Although these microgametes appeared to bud off from the microgamont, the identity of the sausage-like structures could not be determined. Furthermore, extra large gamont-like cells with an unknown role were detected (30 × 35 μm; Figure 4I).

**Figure 5:**
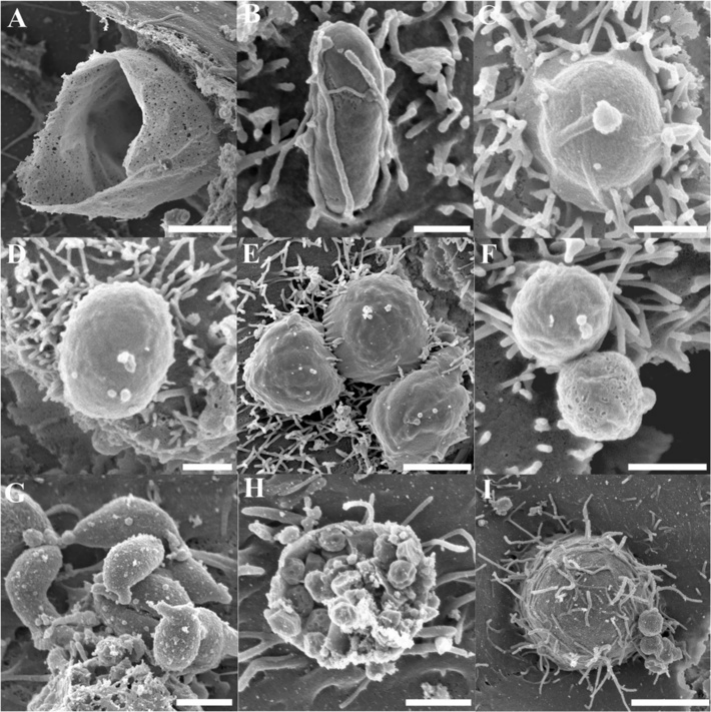
**Scanning electron micrographs of various** ***Cryptosporidium*** **stages identified from** ***Cryptosporidium*** **infected** ***in***-***vitro*** **cell culture showing complementary stages to that seen in biofilms. (A)** Empty oocyst; **(B-D)** Various shaped individual trophozoites; **(E)** Aggregated trophozoites; **(F)** Type I merozoites; **(G)** Type II merozoites; **(H)** Microgamonts; **(I)** Large gamont-like cell. Scale bars = A: 2 μm; B: 0.5 μm; C-D & F-G: 1 μm; E: 2 μm; H: 1.5 μm; I: 4 μm.

To further support and consolidate our identification of *Cryptosporidium* extracellular stages in biofilms, *Cryptosporidium*-infected HCT8 cells were also imaged for comparison. Very similar morphological structures that were observed in biofilms were also observed in cell culture, including empty oocysts with a wide opening and rough membrane (Figure 5A), sporozoites (Figure 5B), rounded trophozoites (both individual and aggregated; Figure 5C-E; 1.5 - 2 μm), free circular shaped type I merozoites (Figure 5F; 1 × 1 μm), free spindle shaped type II merozoites (Figure 5G; 1 × 2 μm), microgamonts with microgametes (Figure 5H; 4 × 3 μm), and large gamont cells (Figure 5I; 5 × 5 μm).

**Figure 6:**
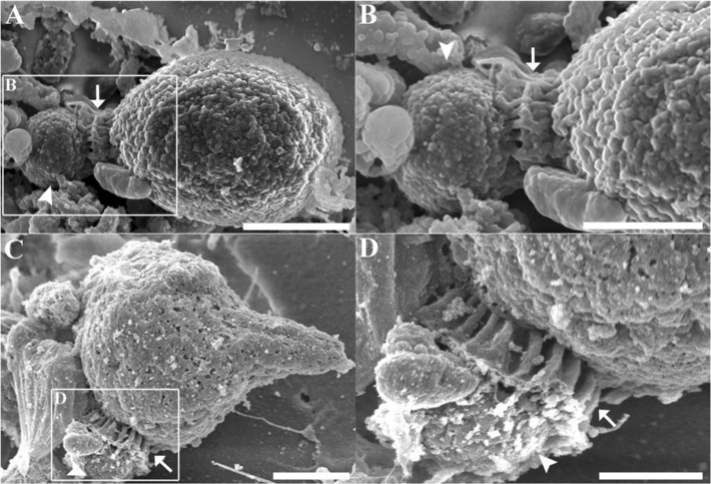
**Parasitophorous vacuole formation. (A)** Scanning electron micrograph showing evidence of parasitophorous vacuole formation by *Cryptosporidium* in a biofilm environment. **(B)** Magnified region depicted in A. **(C)** Parasitophorous vacuole formation by *Cryptosporidium* in HCT8 cell culture, included for comparison. **(D)** Magnified area depicted in **C**. Arrows indicate radial fold and arrowheads indicate dense band area. Scale bars = **A**: 1.5 μm; **B & D**: 1 μm; **C**: 2μm.

In addition to various developmental stages, the formation of a parasitophorous vacuole (PV) by *Cryptosporidium* was observed in biofilm environments (Figure 6A and B). *Cryptosporidium* with PV structures were observed in the biofilm systems from day 1. The morphologies of these closely resembled PVs that were formed by *Cryptosporidium* in HCT8 cell culture (Figure 6C and D), with the presence of radial folds and a button-like dense band area.

### Correlative studies

**Figure 7:**
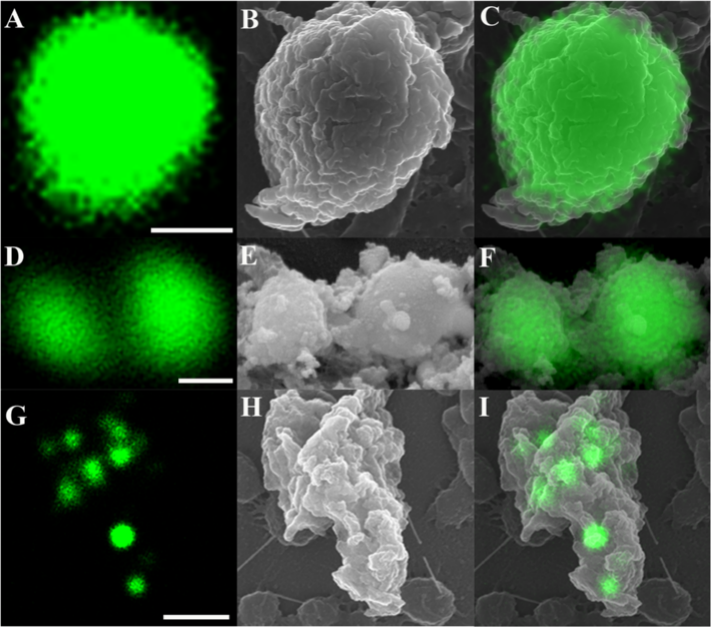
**Direct correlation of** ***Cryptosporidium*** **stages using confocal microscopy (A, D, G) and SEM (B, E, H). (C, F, I)** Superimposed confocal and SEM images; **(A-C)** Single trophozoite; **(D-F)** Free type II merozoites; **(G-I)** Type I meront containing type I merozoites. Scale bars = **A**: 1 μm; **D**: 0.5 μm; **G**: 2 μm.

Correlative studies imaging the same cells by both confocal microscopy and SEM further clearly identified that the extracellular stages observed in flow cell biofilms belonged to *Cryptosporidium*. Several key developmental stages representing both asexual and sexual reproduction were successfully correlated, including trophozoites, meronts, and merozoites. Figure 7 shows structures that intensely expressed Sporo-Glo™ antibody when examined under confocal microscopy were trophozoites (Figure 7A-C) and merozoites (Figure 7D-F). Under confocal microscopy, a group of type I merozoites were also detected (Figure 7G-I). However, SEM revealed that this group of cells were not free merozoites, but rather were residing within a meront that was not fluorescently labelled by Sporo-Glo™ antibody (Figure 7I) and, therefore, not visible in the confocal image.

**Figure 8:**
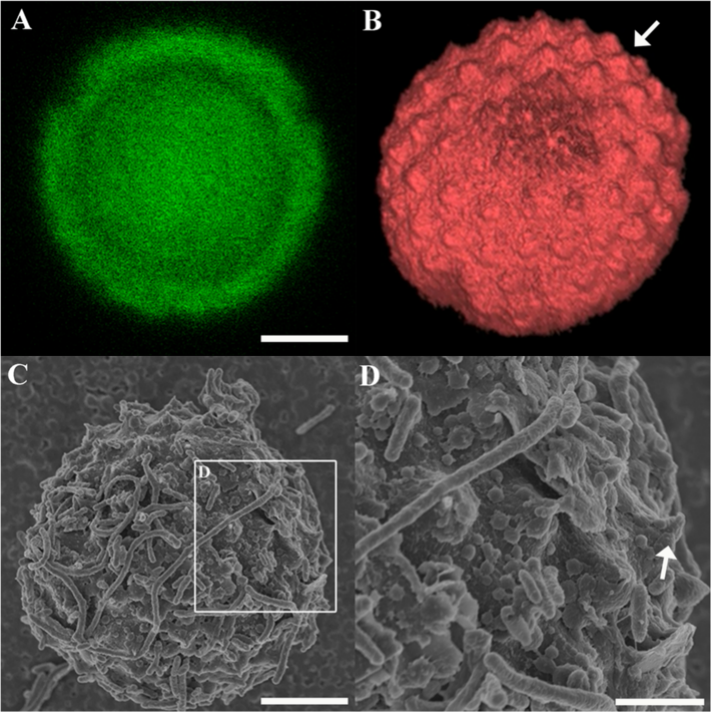
**Correlation of a meront completely enveloped by a PV (arrowhead) under confocal microscopy (A, B) and SEM (C-D). B)** The three dimensional Z-stack images of image **A**. **D)** Magnified area depicted in image **C** showing undulating membrane peaks (arrow). Scale bars = **A** & **C**: 5 μm; **D**: 2 μm.

Throughout the study, confocal microscopy revealed several large membrane-bound cellular structures (Figure 8A). Both the membrane and internal structures were fluorescently labelled. When these image data are presented in 3-dimensions (Figure 8B), an undulated contour membrane structure can be clearly observed (Figure 8B), and this was further confirmed with SEM imaging (Figure 8C and D). These undulations closely resemble the PV outer membrane described by Landsberg and Paperna [23] from *Cryptosporidium* infecting a fish. Based upon the cell size (16 × 16 μm) as observed in this study, these cells were presumed to be meronts.

### Flow cytometry analysis

#### *Cryptosporidium* oocysts in biofilms

After exposure of unexcysted oocysts to *Pseudomonas* biofilms for up to 6 days, no distinct oocyst population could be detected from the flow cell biofilm (data not shown). Conversely, several distinctive fluorescently labelled populations were observed from the waste samples (Additional file 1). These included intact oocyst populations, which indicates, not surprisingly, that not all oocysts were accumulated within the biofilm. For the oocysts that were trapped and released back into the medium flow, it was evident that some of them had undergone excystation within the immature biofilm from as early as day 1 (Additional file 1). This population was similar to the excysted oocyst population seen in the cell-free culture (Additional file 2). As the biofilms matured from day 3 to day 6, it also appeared that more oocysts had undergone excystation (Additional file 1).

Additionally, after both 3 and 6 days, a prominent biofilm population was identified within the flow cytometry profile. This biofilm population was similar to the population found in the biofilm-only controls (Additional file 3), which suggests Crypt-a-Glo™ antibody may label some unknown component in the biofilm. Interestingly, an unknown population that could only be observed in 6 day-old *Cryptosporidium*-exposed biofilms was identified (Additional file 1). The population expressed lower fluorescence intensity but had similar cell sizes to that of intact oocyst populations. The exact identity of this population remains unknown, but we suggest that these may be non-viable oocysts or oocysts that no longer expressed particular surface antigens after prolonged exposure to the biofilm environment.

When similar batches of unexcysted oocysts were introduced into the biofilm-free system, only intact oocyst populations were identified over the 6 day period (Additional file 1). Neither excysted oocysts nor other fluorescent populations such as matrix or unknown populations were detected within the samples. These observations confirmed that oocysts did not excyst in the absence of a biofilm.

### Observation of morphological changes to oocysts in cell-free culture

To further support our observation in the biofilm system, flow cytometry and confocal microscopy were used to observe morphological changes in cultured oocysts during excystation and to correlate this with the observed oocyst populations in *Cryptosporidium*-exposed biofilm samples. Flow cytometry showed that only unexcysted oocysts were present before excystation with acid water (Additional file 2). Immediately after the excystation procedure, a small population of excysted oocysts were identified in the flow cytometry profile (Additional file 2). Following further incubation in maintenance medium for 60 min or 24 h, the amount of fluorescent from excysted oocyst population increased (Additional file 2). Confocal and bright field microscopy revealed populations of oocysts in varying states and exhibiting variable sizes, which coincided well with the flow cytometry profile (Additional file 4).

## Discussion

By using a combination of correlative techniques, including confocal microscopy, SEM and flow cytometry, the findings described here demonstrate that *Cryptosporidium* can not only excyst within aquatic biofilms, but also develop and multiply extracellularly without an epicellular association with a host cell. These stages include sporozoites, trophozoites, large meronts, merozoites, microgamonts, gamont-like cells, and extra-large gamont-like cells. The observation of these extracellular stages has served to emphasise that the extracellular capability of *Cryptosporidium* cannot be disregarded.

Traditional methods of confocal microscopy and immunolabelling were performed to reveal the subsequent fate of *Cryptosporidium* after exposure to biofilms. Overall, we showed that excystation occurred from day 1. Similar to other cell-free cultures studies [14,16], aggregated trophozoites were commonly observed in the biofilms. These trophozoites may have fused together by a syzygy-like process, as described previously by Borowski et al. [13] and Hijjawi et al. [14].

By day 6, developmental stages of *Cryptosporidium* were much more prominent and easily identified, including the presence of a gamont-like stage. Since this stage has not been commonly observed in the *Cryptosporidium* life cycle, Woods and Upton [24] suspected that the presence of this stage in both cell-free and *in*-*vitro* cultures was due to contaminating debris or fungal infection, resembling *Bipolaris australiensis* and *Colletotrichum acutatum*. However, the intense immunofluorescent labelling of the internal structures in this case, which resembled merozoites or trophozoites, counters this argument. Our observations suggest that the role of this stage is to generate trophozoites and merozoites so that more new oocysts can be produced without host encapsulation.

In addition to our confocal observations, SEM analysis not only revealed detailed surface structural data for several stages, such as sporozoites, trophozoites, free and encapsulated type II merozoites, type I and II meronts, and microgamonts, but also demonstrated that *Cryptosporidium* has the ability to form a parasitophorous vacuole (PV) independent of a host. The formation of a PV within biofilms is intriguing as a PV has not previously been reported in cell-free cultures [14,17,18]. This may be due to the fact that it is difficult to identify PVs using optical microscopy and hence the formation of a PV in cell-free culture has gone undetected. The PVs observed here (in both biofilm systems and HCT8 cell cultures) were very similar to those observed in previous *in*-*vitro* and *in*-*vivo* studies [23,25-28], but these studies also believed that the undulated membrane was derived from the host microvillus. The formation of a PV in our host free system is consistent with the proposal by Pohlenz et al. [29] that *Cryptosporidium* does not require host encapsulation to form a PV.

Besides PV formation, the identification of large meronts and extra large gamont-like stages suggests that biofilms can modify and influence *Cryptosporidium* developmental stages in aquatic environments. Although the identity and the role of extra large gamont-like cells are still unknown, similar cell sizes and morphologies have been observed in the closely related gregarines [30-32]. Therefore, it is suspected that they may also have a role in producing infective stages of *Cryptosporidium* in biofilms. In addition, the correlative imaging of a large meront showed that apart from type I merozoites located within the cell producing fluorescence, no other part of the parent cell fluoresced. The inability to label these gamont stages and meront walls with Sporo-Glo™ antibody suggests surface epitopes that bind Sporo-Glo™ were not expressed by these stages in the biofilms. The observations made in this study shows that Sporo-Glo™ antibody does not label all extracellular stages in biofilms and a new antibody that targets more of the *Cryptosporidium* developmental stages is needed to further investigate the life cycle of *Cryptosporidium* in biofilms.

Similar to the observations of Wolyniak et al. [6], our flow cytometry analysis also revealed that oocyst populations were observed throughout the experiment. However, the flow cytometry data further demonstrate that these oocysts were actually present as two different oocyst populations - excysted and intact oocysts. In addition, parallel confocal microscopy observations of cell-free culture experiments confirmed that the excysted oocyst populations consisted of excysted oocysts of variable sizes, and not degraded oocysts. Furthermore, a previous study by King et al. [21] demonstrated that the hydrodynamics of the flow resulted in empty oocysts collapsing, causing them to be much smaller and more refractive. Nevertheless, the absence of excysted oocyst populations in the biofilm-free samples demonstrates that *Cryptosporidium* does not excyst in the absence of a biofilm in an artificial aquatic environment.

## Conclusions

The observation of extracellular developmental stages in this study further supports suggestions that classification of *Cryptosporidium* as an obligate intracellular apicomplexan may require revision [14,15,19,33,34]. As this study used diluted tryptic soy broth medium in our *Pseudomonas* biofilm system, the public health significance of these extracellular stages in relation to waterborne outbreaks has to be determined. These risks will be dependent on a range of factors including whether development can occur in natural biofilms, if the lifecycle can be completed through to oocyst stage, the infectivity of these extracellular stages, and their susceptibility to disinfectants. Undoubtedly, the interaction between *Cryptosporidium* and biofilms warrants further investigation.

## Methods

### *Cryptosporidium parvum* oocysts preparation

*Cryptosporidium parvum* cattle isolate used in the study was originally obtained from the Institute of Parasitology, University of Zurich. The use of this isolate was approved by the Murdoch University Institutional Biosafety Committee (Australia). Fresh oocysts were purified and obtained by passage through infected ARC/Swiss mice as described by Meloni and Thompson [35]. These purified oocysts were stored in 1 × phosphate buffered saline (PBS) with antibiotics (10,000 U penicillin G and 0.01 g streptomycin; Sigma) at 4°C before use. All oocysts used in this study were not more than 4 weeks old and similar batches of oocysts were used for parallel experimental controls. Oocysts used in this study were pre-treated with 2% household bleach in distilled water for 20 min at room temperature. They were then pelleted at 3500 rcf and resuspended with 200 μl of sterile 1 × PBS. This animal work was reviewed and approved by the Animal Ethics Committee of Murdoch University, Australia (Permit number: R2310/10).

### Biofilm flow cell system

Wild type strain *Pseudomonas aeruginosa* (PA01) was used to produce biofilms and was maintained on *Pseudomonas* agar prior to incubation into flow cell systems. The flow cell biofilm system was operated as described previously [19]. Three experimental conditions were used, with each replicated 3 times:

1. *Cryptosporidium*-exposed biofilm: oocysts were injected into the influent medium and the flow initiated and continued (60 ml h^−1^) for one, three or six days.
2. Biofilm-free control. No biofilm was developed. Decontaminated oocysts were injected into the flow system and maintained as above.
3. Biofilm-only control. Biofilms were grown in the flow system without the introduction of *Cryptosporidium* oocysts.

### Biofilm dispersion

*Cryptosporidium*-exposed, biofilm-free and biofilm-only systems were dispersed with 500 nM of sodium nitriporusside (nitric oxide) overnight (Sigma, USA). This nitric oxide was pumped into the flow cell overnight and undisturbed for 24 h. After this, these flow cells were gently washed with 1 × PBS several times to completely detach the biofilms from the flow cell surface [36]. The cell suspensions were further washed several times with sterile 1 × PBS to remove any residual nitric oxide and immediately post–fixed with 2.5% paraformaldehyde in sterile 1 × PBS for 20 min at 4°C. The cells were pelleted at 3500 *g* for 10 min and resuspended in sterile 1 × PBS to a final volume of 400 μl. Similarly, the cells (unattached biofilms/bacteria/*Cryptosporidium*) in the effluent/waste bottle were also collected by centrifuging at 4°C and 1000 rpm for 1 hour and post–fixed with 2.5% paraformaldehyde in sterile 1 × PBS for 20 min at 4°C. The fixed sample was resuspended to a final volume of 2 ml. Prior to microscopic and flow cytometry analyses, an aliquot of flow cell (100 μl) and effluent (500 μl) samples were subjected to qPCR analysis for determination of *Cryptosporidium* number as described previously [19].

### Cell-free culture

Cell-free culture was performed to follow morphological changes in oocysts during the excystation process. *Cryptosporidium* oocysts (4 × 10^6^) were excysted with excystation media (0.5% trypsin-EDTA, pH 2.5) for 30 min at 37°C. An aliquot (1 × 10^6^) of solution was immediately post–fixed with 2.5% paraformaldehyde in sterile 1 × PBS for 20 min at 4°C. The cells were pelleted and resuspended to a final volume of 200 μl. This sample was stored at 4°C for further flow cytometry analysis and is hereafter referred to as excysted oocysts. The remaining excysted oocysts were kept in maintenance medium for a further 60 min or 24 h at 37°C. These samples are referred to as 60 min and 24 h culture samples respectively. The maintenance medium consisted of 10% RPMI media, 0.03 g l^−1^ glutamine, 0.3 g l^−1^ sodium bicarbonate, 0.02 g l^−1^ bovine bile, 0.1 g l^−1^ glucose, 25 μg g l^−1^ folic acid, 100 μg l^−1^ 4-aminobenzoic acid, 50 μg g l^−1^ calcium pantothenate, 875 μg g l^−1^ ascorbic acid, 1% fetal calf serum (FCS), 0.36 g l^−1^ HEPES buffer, 10,000 U penicillin G and 0.01 g l^−1^ streptomycin, adjusted to pH 7.4.

### HCT8 cell line infections

For direct comparison with the *Cryptosporidium* extracellular stages in biofilms, human colon carcinoma cell line (HCT-8) was routinely passaged and maintained in RPMI 1640 medium (pH 7.2) containing 2 g L^−1^ sodium bicarbonate, 0.3 g L^−1^ L-glutamine, 3.574 g L^−1^ HEPES buffer (15 mmol L^−1^) and 10% heat inactivated FCS, at 5% CO_2_ and 37°C. HCT8 cells infected with *Cryptosporidium* were prepared as previously described in Borowski et al. [13]. The infected cultures were sampled and processed for SEM analysis at 6, 24, 55, and 120 h post inoculation.

### Immunolabelling

#### Developmental stage-specific antibody

Confocal laser scanning microscopy was used to monitor the subsequent development of *Cryptosporidium* in aquatic biofilms. Currently, Sporo-Glo™ (Waterborne Inc.) is the only commercially available antibody which specifically targets *Cryptosporidium* developmental stages [16,37]. The binding pattern of Sporo-Glo™ antibody was previously studied in detail and it was shown that the antigen is not expressed or cannot be detected until the oocysts have begun excystation [19,38]. Additionally, intense fluorescence expression can be detected from several developmental stages [16,19,38]. An aliquot of dispersed biofilm suspension (20 μl) was fixed with 2.5% paraformaldehyde in PBS for 20 min at 4°C and immunolabeled with quantum dot (#Q-11621MP, Invitrogen) conjugated Sporo-Glo™ antibody as described previously [19].

#### Oocyst-specific antibody

As Sporo-Glo™ antibody is only expressed after excystation, it is not suitable for tracking the changes to oocyst morphology [19,38]. As such, the changes of oocyst morphology in cell-free cultures, *Cryptosporidium*-exposed biofilms, and biofilm-free systems were determined by labelling with the *Cryptosporidium* oocyst-specific antibody, Cy5-Crypt-a-Glo™ (Waterborne, Inc), which is constantly expressed on the oocyst wall regardless of excystation status. All samples (200 μ1) including unexcysted *Cryptosporidium* oocysts (1 × 10^6^), *Cryptosporidium*-exposed biofilms (flow cell biofilms and waste samples), biofilm-free controls and *Cryptosporidium* cell-free cultures were immunolabeled with Crypt-a-Glo™ antibody (5.0 μg ml^−1^) at room temperature in a dark room for 30 min. The cells were then washed twice with 1 × PBS for 5 min each and pelleted at 3500 rcf for 10 min. Except for cell-free culture samples, all other samples were resuspended to a final volume of 200 μl. Cell-free culture samples were resuspended in 400 μ1 of sterile 1 × PBS and an aliquot of each cell-free culture sample (200 μ1: 5 × 10^5^ oocysts) was taken and processed for morphological analysis by confocal microscopy. The remaining samples were analysed by flow cytometry on the same day.

During flow cytometry analysis, an unknown population was detected in 3 and 6 day-old *Cryptosporidium*–exposed biofilms. Previous studies had shown that biofilm matrix tends to trap and prevent the penetration of antibodies into biofilms [39,40]. Therefore, to determine whether this population could also be detected in the biofilm-only controls, immunolabelling with Cy5-Crypt-a-Glo™ antibody as described above was performed on the 6 day-old biofilm-only sample.

### Confocal microscopy

For examination of *Cryptosporidium* biofilm samples or cell-free cultures by confocal laser scanning microscopy, an aliquot of the immunolabeled suspension was immobilised and attached to coverslips using 0.01% poly-L-lysine (Sigma, USA). The samples were then analysed directly by confocal microscopy (Leica SP2 inverted) using excitation/emission wavelengths of 488/525 nm (quantum dots) or 649/688 nm (Cy5). Both confocal and bright field images were acquired simultaneously. For correlative analyses, serial sections on the *xy* plane were obtained at 0.44 μm along the *z*-axis for meront stage and the three dimensional Z-stack images were constructed using Volview™.

### Scanning electron microscopy

An aliquot (20 μl) of dispersed flow cell biofilm sample was further fixed in an equal volume of 5.0 % glutaraldehyde in sterile 1 × PBS solution. The samples were immobilised and attached onto 0.01% poly-L-lysine (Sigma, USA) coated round coverslips (12 mm). To prevent sample dehydration at room temperature, the sample was left to stand in a humidified box for 30 min. For the infected cell line, RPMI medium was removed and the coverslips with adherent cells were fixed in 2.5% glutaraldehyde in sterile 1 x PBS solution. These samples were then dehydrated through a series of ethanol concentrations and critical point dried as described in Borowski et al. [13].

Dried coverslips were mounted on stubs with carbon tape. These were then coated with 4 nm carbon and 5 nm platinum for stable, high resolution imaging using the in-lens secondary electron detector at an accelerating voltage of 3 kV (Zeiss 55 VP field emission SEM).

### Correlative study

An SEM finder grid was used to locate the same fluorescent cells from the confocal, in the SEM. The SEM grid finder pattern was made on a sample mould that could be seen using both confocal and SEM, allowing for the establishment of directly correlative studies. The SEM finder grid pattern was transferred from the mould onto a 12 mm circular coverslip as described by Powell et al. [41]. Essentially, an SEM finder grid was placed flat on the 12 mm circular coverslip and sputter-coated with Pd/C for 3 min to produce a clear outline of the SEM finder grid visible under both confocal microscopy and SEM.

For this experiment, Cel-Tak™ coated round coverslips (12 mm) were used to immobilise and adhere dispersed flow cell biofilm samples (20 μl). Again, the sample was left to stand in a humidified box for 15 min. During the incubation period, a clean sterile square coverslip (22 × 22 mm) was placed onto the square coverslip holder in preparation for confocal analysis. The SEM coverslip was removed from the humidified box and placed onto the square coverslip. As we were using inverted confocal microscope, the SEM coverslip was gently turned over after 15 min of incubation so that the side with the experimental sample was facing down towards the square coverslip. After analysis by confocal microscopy and the location of the cells of interest had been recorded, the sample was fixed in 2.5% glutaraldehyde in sterile 1 × PBS and immediately processed for SEM by freeze drying.

Prior to freeze drying, the coverslip was briefly rinsed in 150 mM ammonium acetate and blotted on filter paper to remove the excess liquid. The sample was then rapid frozen in liquid nitrogen slush and freeze dried (Emitech K775X turbo pumped freeze drying system) in a step wise fashion (−120°C to −65°C over 14 h; −65°C to +25°C over 10 h; hold at 25°C for 24 h). The samples were then mounted on stubs with carbon tape and coated with 4 nm carbon and 5 nm platinum. It was observed that the Pt/C pattern could be visualised at 10 kV with the SE2 detector. Although the in-lens detector provides better image resolution at 3 kV, no grid pattern underneath the sample could be observed at this lower voltage. Therefore, 10 kV was used, with the SE2 and in-lens detector selected for grid and sample observation respectively.

### *Cryptosporidium* Descriptions

Due to the immense numbers of bacteria relative to *Cryptosporidium* within the biofilm, several attempts at cutting thin (~100 nm) sections for transmission electron microscopy observation failed to locate life stages within the masses of biofilm at this scale. Therefore, C*ryptosporidium* developmental stages as described in this study were correlated with, and identified based on previous detailed morphological descriptions in *in*-*vivo*, cell-free, and *in*-*vitro* systems [13,14,16,28,38,42-44].

### Flow cytometry

All samples were analysed using flow cytometry (FACSCalibur, Becton Dickinson, Australia). Logarithmic signals were used for all parameters and the forward light scatter detector was set at E00 for all assays. The forward scatter detector was set to 396 V and the red fluorescent detector to 597 V.

To investigate the possibility of *Cryptosporidium* excystation in the biofilm system and cell-free culture, gating was used to discriminate and differentiate *Cryptosporidium* oocyst populations from mixed populations. The acquisition gate was determined based on the fluorescence (intensity) and forward scatter characteristics of Cy5- Crypt-a-Glo™^−^labelled pure bacteria and *Cryptosporidium* oocysts (unexcysted/excysted). In addition, cell free culture samples were also used to identify excysted oocyst populations. As such, two different populations were similarly used to differentiate gated areas for each of the physiological states of oocysts - intact and excysted. The collected data was analysed using FlowJo software (TreeStar).

## Additional files

Additional file 1:
**Flow cytometric profiles of oocyst populations from 1, 3 and 6 day-old** ***Cryptosporidium*** **-exposed biofilms (A-C) and biofilm free (D-F) samples.** All waste samples were labelled with Crypt-a-Glo™ monoclonal antibody. Additional file 2:
**Flow cytometric profiles of oocyst populations from cell-free culture.** A) Unexcysted oocysts; B) immediately excysted oocysts; C) 60 min after excystation; and D) 24 h after excystation. All samples were stained with Cy5-labelled Crypt-a-Glo™ monoclonal antibody. Additional file 3:
**Flow cytometric profiles of 6 day-old biofilm-only controls.** (A) Unlabelled control; (B) Crypt-a-Glo™ labelled control. Additional file 4:
**Superimposed confocal and bright field images of oocyst populations 60 minutes after excystation, which are stained with Crypt-a-Glo™ antibody.** (A) Intact oocyst. Internal structure visible within the oocyst is evidence of an intact oocyst complete with sporozoites and residual bodies. (B) Internal contents of an oocyst excysting into the surrounding environment. (C-F) Empty oocysts undergoing a series of morphological changes and size reduction. Scale bars = A: 5 μm; B: 2.5 μm; C =5 μm; D-E: 2.5 μm; F: 2 μm.
