# Supplementary figures and images for: Extracellular excystation and development of Cryptosporidium: tracing the fate of oocysts within Pseudomonas aquatic biofilm systems
Source: BMC Microbiol. 2014 Nov 18;14:281. doi: 10.1186/s12866-014-0281-8 (PMC4236811; doi:10.1186/s12866-014-0281-8)

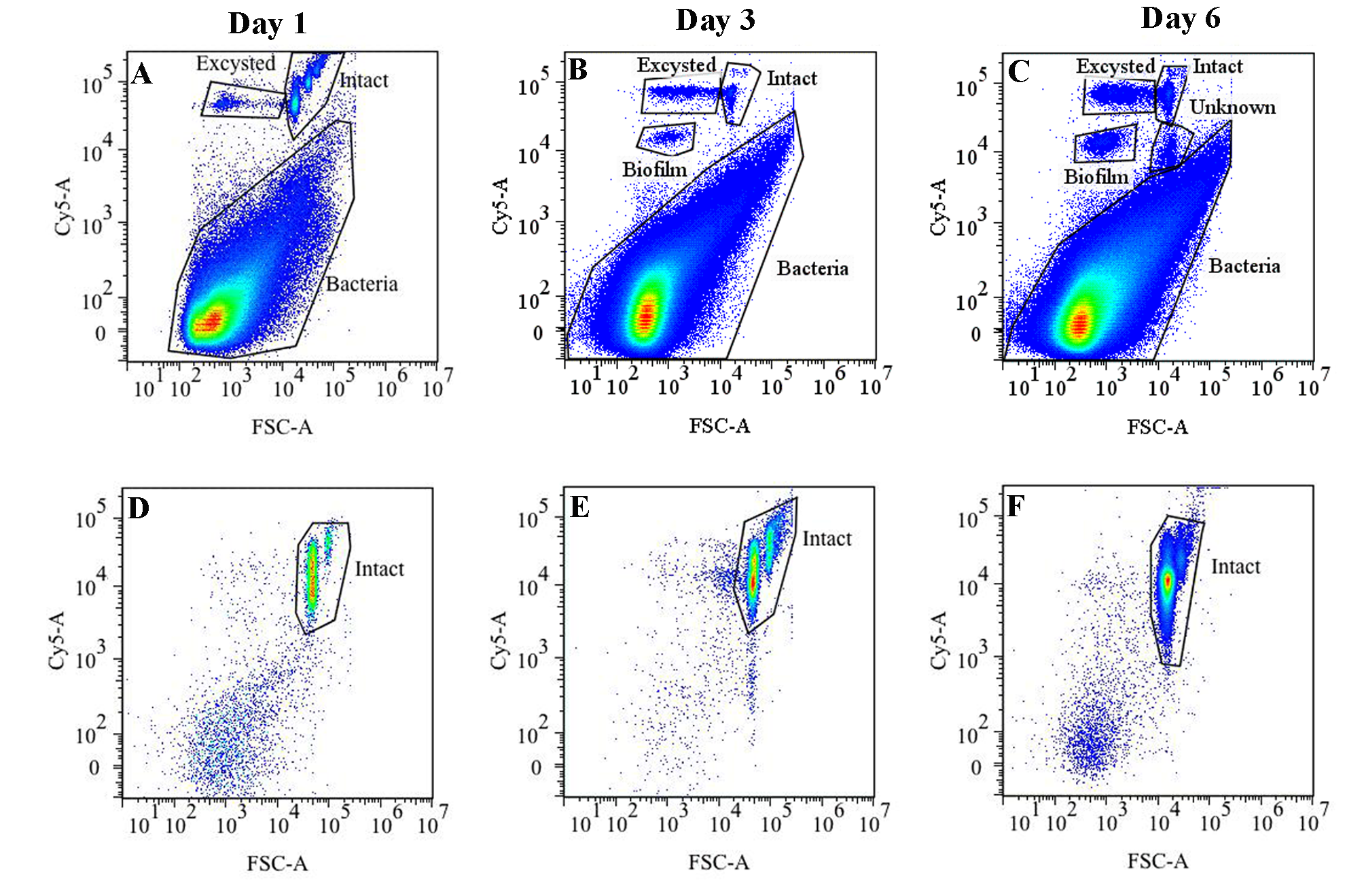

Supplement: Additional file 1: — Flow cytometric profiles of oocyst populations from 1, 3 and 6 day-old Cryptosporidium -exposed biofilms (A-C) and biofilm free (D-F) samples. All waste samples were labelled with Crypt-a-Glo™ monoclonal antibody. [file 12866_2014_281_MOESM1_ESM.tiff]

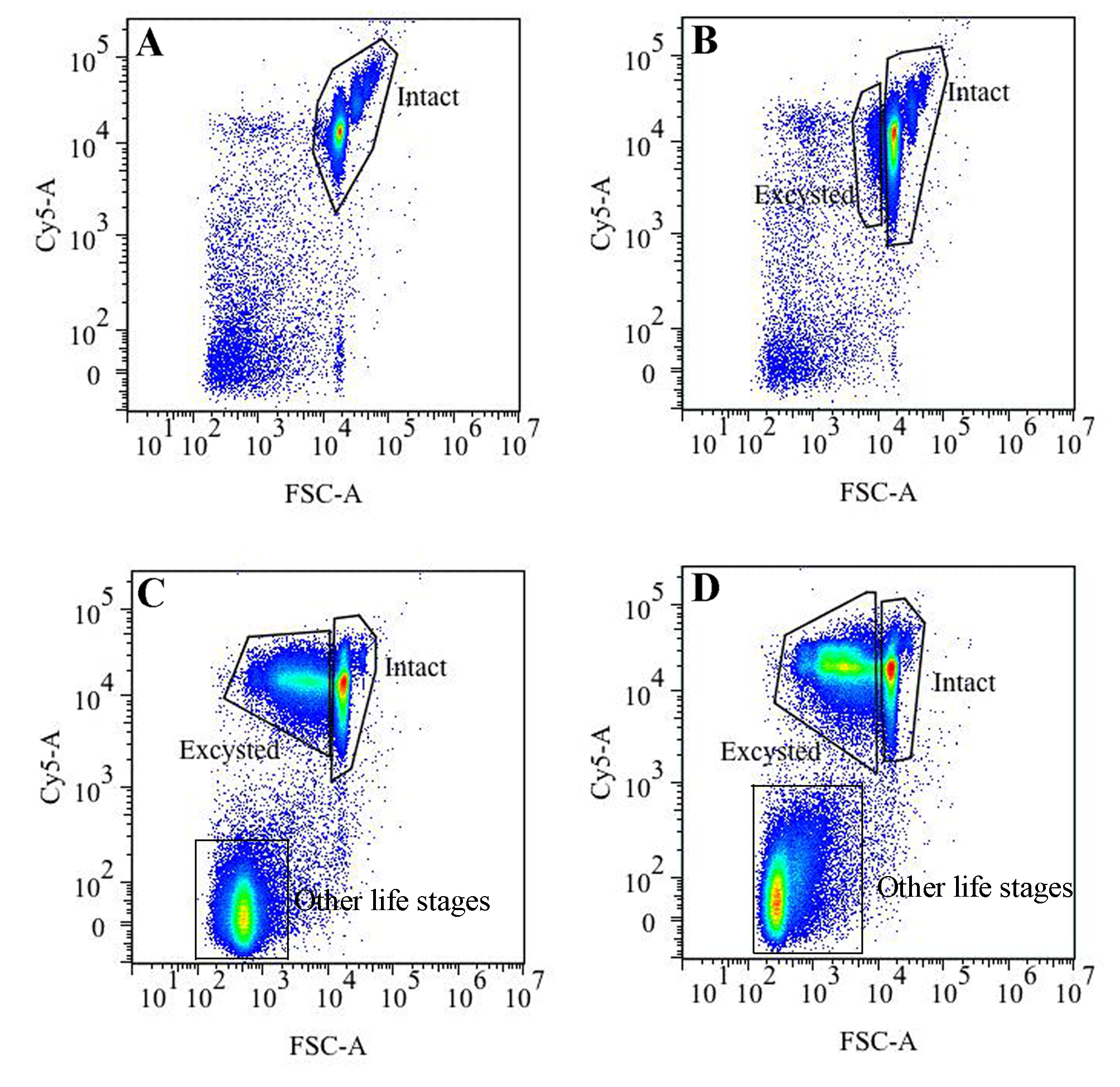

Supplement: Additional file 2: — Flow cytometric profiles of oocyst populations from cell-free culture. A) Unexcysted oocysts; B) immediately excysted oocysts; C) 60 min after excystation; and D) 24 h after excystation. All samples were stained with Cy5-labelled Crypt-a-Glo™ monoclonal antibody. [file 12866_2014_281_MOESM2_ESM.tiff]

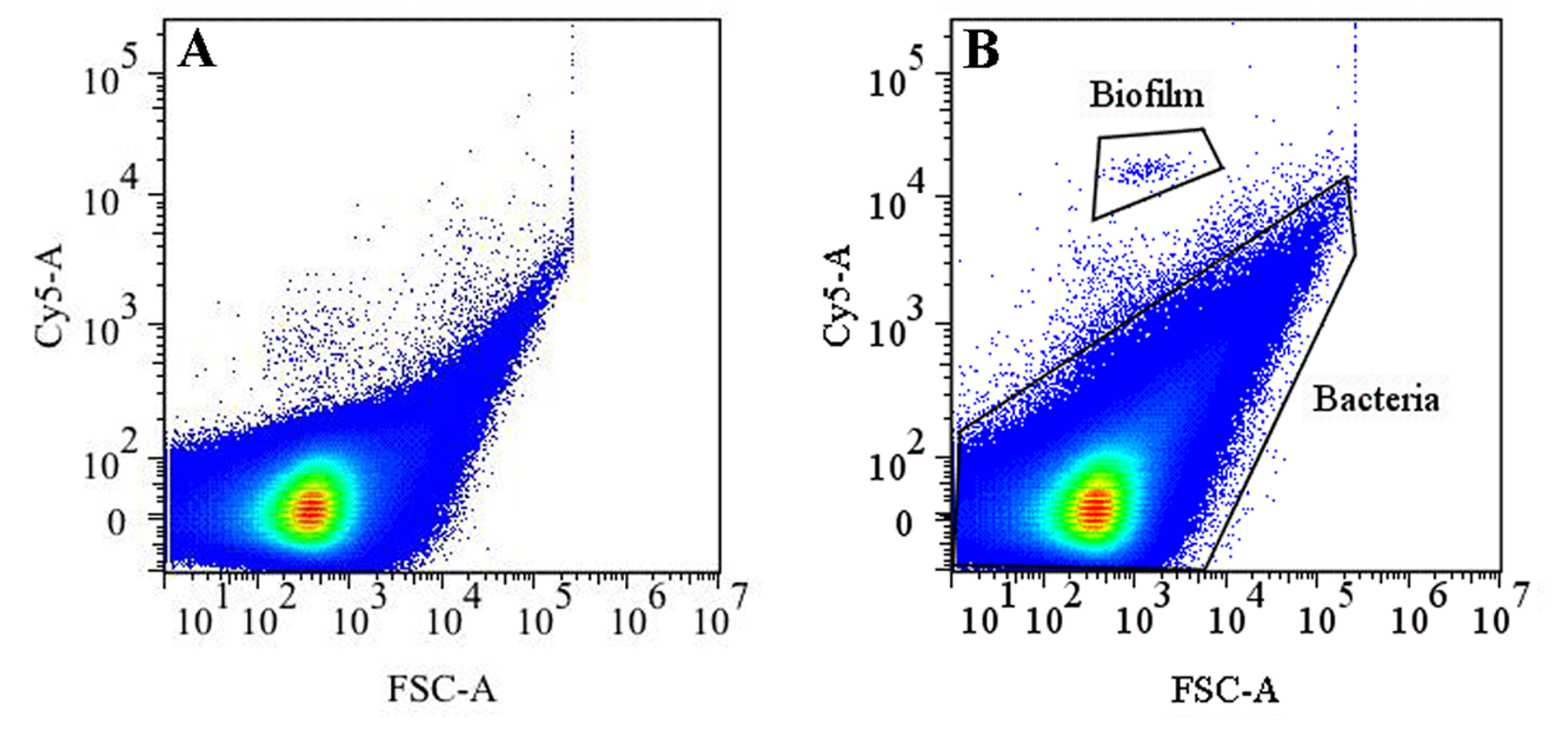

Supplement: Additional file 3: — Flow cytometric profiles of 6 day-old biofilm-only controls. (A) Unlabelled control; (B) Crypt-a-Glo™ labelled control. [file 12866_2014_281_MOESM3_ESM.tiff]

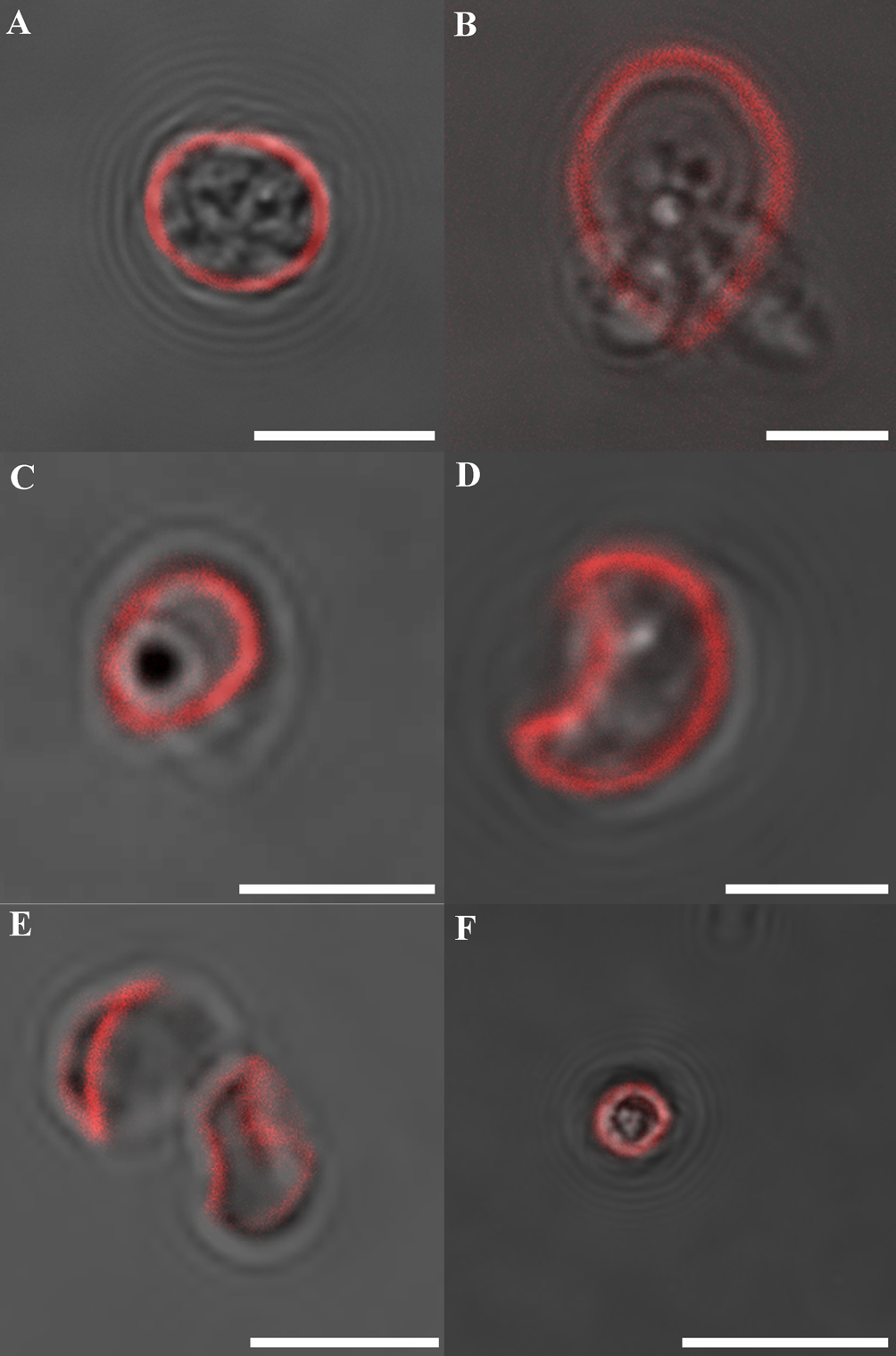

Supplement: Additional file 4: — Superimposed confocal and bright field images of oocyst populations 60 minutes after excystation, which are stained with Crypt-a-Glo™ antibody. (A) Intact oocyst. Internal structure visible within the oocyst is evidence of an intact oocyst complete with sporozoites and residual bodies. (B) Internal contents of an oocyst excysting into the surrounding environment. (C-F) Empty oocysts undergoing a series of morphological changes and size reduction. Scale bars = A: 5 μm; B: 2.5 μm; C =5 μm; D-E: 2.5 μm; F: 2 μm. [file 12866_2014_281_MOESM4_ESM.tiff]
